# Comparing and phylogenetic analysis chloroplast genome of three *Achyranthes* species

**DOI:** 10.1038/s41598-020-67679-y

**Published:** 2020-07-02

**Authors:** Jingya Xu, Xiaofeng Shen, Baosheng Liao, Jiang Xu, Dianyun Hou

**Affiliations:** 1 Agricultural College, Henan University of Science and Technology, Luoyang, China; 2 The Luoyang Engineering Research Center of Breeding and Utilization of Dao-Di Herbs, Luoyang, China; 3 Institute of Chinese Materia Medical, China Academy of Chinese Medical Sciences, Beijing, China

**Keywords:** Molecular biology, Plant sciences

## Abstract

In this study, the chloroplast genome sequencing of the *Achyranthes longifolia*, *Achyranthes bidentata* and *Achyranthes aspera* were performed by Next-generation sequencing technology. The results revealed that there were a length of 151,520 bp (*A. longifolia*), 151,284 bp (*A. bidentata*), 151,486 bp (*A. aspera*), respectively*.* These chloroplast genome have a highly conserved structure with a pair of inverted repeat (IR) regions (25,150 bp; 25,145 bp; 25,150 bp), a large single copy (LSC) regions (83,732 bp; 83,933 bp; 83,966 bp) and a small single copy (SSC) regions (17,252 bp; 17,263 bp; 17,254 bp) in *A. bidentate, A. aspera* and *A. longifolia*. There were 127 genes were annotated, which including 8 rRNA genes, 37 tRNA genes and 82 functional genes. The phylogenetic analysis strongly revealed that *Achyranthes* is monophyletic, and *A. bidentata* was the closest relationship with *A. aspera and A. longifolia*. *A. bidentata* and *A. longifolia* were clustered together, the three *Achyranthes* species had the same origin, then the gunes of *Achyranthes* is the closest relative to *Alternanthera*, and that forms a group with *Alternanthera philoxeroides*. The research laid a foundation and provided relevant basis for the identification of germplasm resources in the future.

## Introduction

*Achyranthes* L. is the herbaceous or subshrub plant, which distributed in the tropical and subtropical regions of both hemispheres, including 15 species. Among them, 3 species are found in China, that are *Achyranthes bidentata, A. aspera and A. longifolia.* Of which*, A. bidentata* is widely distributed in China, especially in Henan province^1^. The roots of *A. bidentata* is used as an important traditional Chinese medicine (TCM) to treat bones fracture and osteoporosis^1–3^, and it is also used in the treatment of arthritis^4^ or enhance immunity^5^. Besides, it is a medicinal herb with the property of activating blood to regulate menstruation and it can be used to tonify the liver and kidney^6^. Even it is used as the medicine of anti-cancer, anti-inflammatory^7, 8^. The main active ingredients include polysaccharides^9^, saponins^10^, peptides, organic acids and other substances in *A. bidentata*. Because of these secondary metabolites, *A. bidentata* has a variety of physiological activities, which makes it have higher utilization value. The function of *A. longifolia* is similar to that of *A. bidentate*. *A. aspera* is a traditional herbal medicine, is widely distributed in India^11^, also distributed in Hunan of China. The roots was used as the medicine of anticancer^12^, antiarthritic^13^, anti-herpes virus^14^ and antifertility^15^.

Chloroplast is a characteristic organelle in green plant cells, and is the major site for photosynthesis of cells. The chloroplast has its own DNA and genetic system. The chloroplast plays an indispensable role in the evolution of plants^16–18^. As an important research object in the field of molecular evolution, phylogeny, and molecular markers, the chloroplast genome sequencing technology has been widely used in the phylogenetic research of various plant groups^19^.

With the acquisition of chloroplast genome sequence information of tobacco^20^, the structural characteristics of chloroplast genome were revealed, more and more plants have obtained corresponding chloroplast genome information^21–24^. However, there are fewer reports on medicinal plants^25, 26^. According to current research reports, the chloroplast genome of angiosperms generally has a highly conserved quadripartite structure with a length of 120–180 kb, including a small single-copy (SSC) region with a length of 16–27 kb, a large single-copy (LSC) region with a length of 80–90 kb, and a pair of inverted repeat regions (IRs)^27–29^.

At present, most of the researches on *Achyranthes* species are focused on its pharmacological activities and chemical components in worldwide, while the research were less common in the comparing and phylogenetic analysis chloroplast genome of *Achyranthes* species. By comparing the chloroplast genome sequences of plants, we can clearly observe the differences among the genomes of different species at the molecular level, and use them as the basis for species division and identification. The chloroplast genome of *A.bidentata* was reported in research of Park^30^ and Li^31^ Park et al. found that the chloroplast genome of Korean *A.bidentata* has the same structural characteristics of angiosperms, while there were the same results of Hubei *A.bidentata* in the research of Li^31^. With the emergence of chloroplast genome sequence, chloroplast genome is also expected to help solve the deeper system development branch. The phylogenetic analysis of chloroplast genome sequence was used to evaluate the evolutionary relationship among species now. In this study, the complete chloroplast genome sequences information of *A. bidentate*, *A. longifolia* and *A. aspera* were obtained by sequencing the whole chloroplast genome, and comparative analyses of their structure and function. All of these will provide valuable reference information for species evolution and phylogeny of *Achyranthes*, and provide new reference for the identification and development of plant resources in the future.

## Results

### *Achyranthes* Chloroplast (CP) Genome structure and content

**Figure 1:**
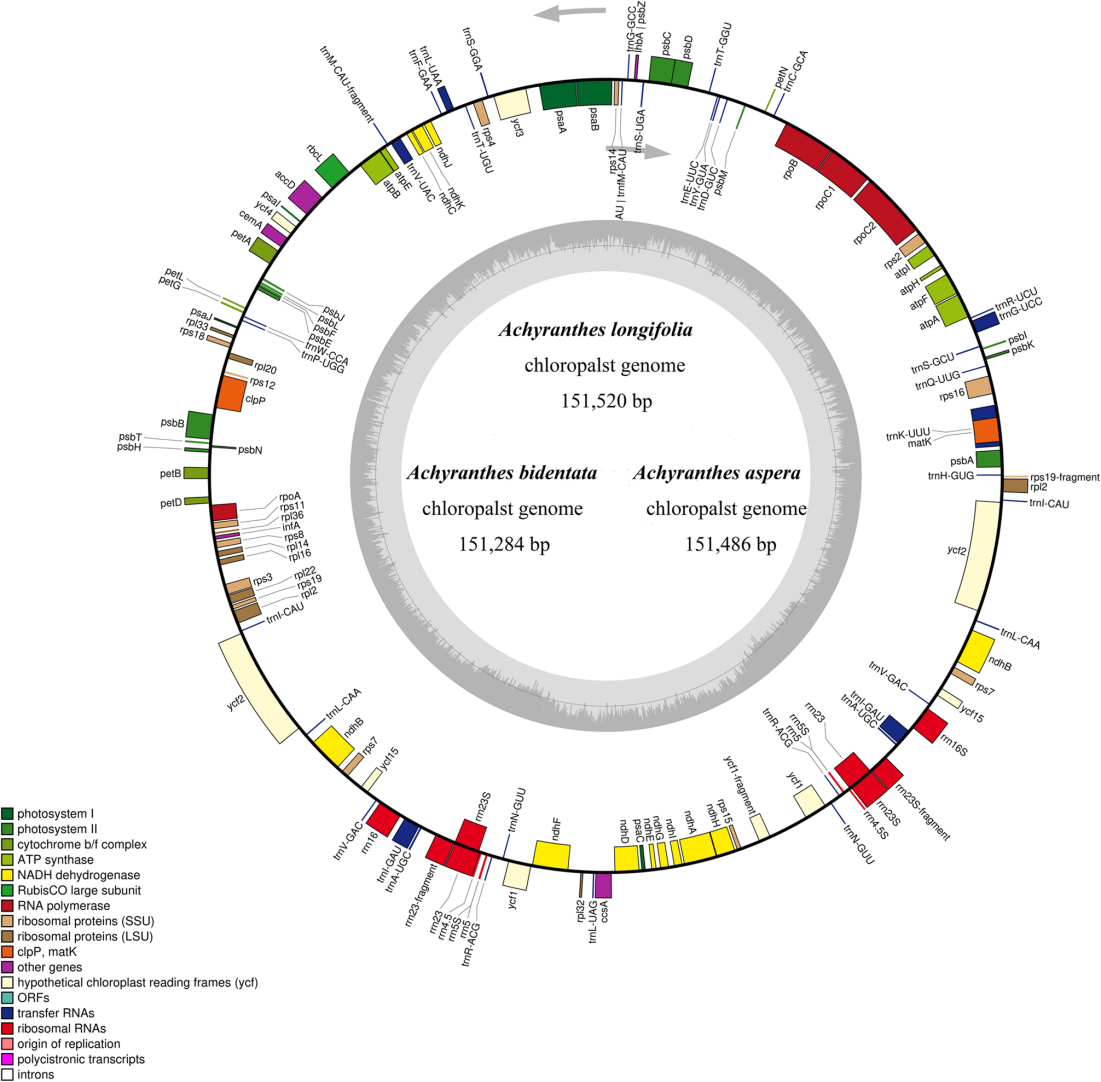
Gene map of the *Achyranthes* chloroplast (CP) genome. Genes drawn inside the circle are transcribed clockwise, genes outside are transcribed counter clockwise. Different colors encode genes belonging to different functional groups. The area is darker gray and lighter gray in the inner circle corresponds GC content and AT content, respectively.

In this study, the whole chloroplast genome sequence of 3 *Achyranthes* species were obtained by sequencing and submitted to the NCBI database with the GenBank accession number MN953049 (*A. longifolia*), MN953050 (*A. bidentata*), MN953051 (*A. aspera*). Then these sequences were analyzed by various means. In this study, in order to validate the assembly sequences of Chloroplast genome of the three *Achyranthes* species, the junction sequences of LSC-IRb, IRb-SSC, SSC-IRa and IRa-LSC regions from three species were amplified and compared with assembly sequences. The results showed that the junction sequences of PCR amplification and assembly sequences were consistent up 99% or more. The results also indicated that the assembly sequences are accurate and reliable. The partial Blast results and DNA peak map of junction sequences was listed in Table S1. The results show that the chloroplast genome of them had a typical quadripartite structure (Fig. 1).

The results also indicated that the complete chloroplast genome had a length of 151,520 bp (*A. longifolia*), 151,284 bp (*A. bidentata*), 151,486 bp (*A. aspera*), respectively (Fig. 1).The structure of chloroplast genome included a small single-copy (SSC) region, a large single-copy (LSC) region and two inverted repeat (IR) regions. The length of the LSC region was 83,966 bp (*A. longifolia*), 83,732 bp (*A. bidentata*), 83,933 bp (*A. aspera*), respectively. Then, the length of the SSC region was 17,254 bp (*A. longifolia*), 17,252 bp (*A. bidentata*), 17,263 bp (*A. aspera*), respectively. Lastly, the IR region had a length distribution of 25,150 bp (*A. longifolia*), 25,150 bp (*A. bidentata*), 25,145 bp (*A. aspera*).

The GC content was 34.1–34.2% in LSC region. The GC content was 30% in SSC region, and the GC content was 42.5% in the IR regions. Thus, the GC content was the lowest in the SSC region. In addition, the total GC content was 36.4% (*A. longifolia*), 36.5% (*A. bidentata*), 36.5% (*A. aspera*), respectively (Table 1).

**Table 1:** Summary of complete chloroplast genomes for three *Achyranthes* species.

Species	*A. longifolia*	*A. bidentata*	*A. aspera*
**LSC**			
Length (bp)	83,966	83,732	83,933
G+C (%)	34.1	34.2	34.2
Length (%)	55.4	55.4	55.4
**SSC**			
Length (bp)	17,254	17,252	17,263
G+C (%)	30.0	30.0	30.0
Length (%)	11.4	11.4	11.4
**IR**			
Length (bp)	25,150	25,150	25,145
G+C (%)	42.5	42.5	42.5
Length (%)	16.6	16.6	16.6
**Total**			
Length (bp)	151,520	151,284	151,486
G+C (%)	36.4	36.5	36.5

The gene content and sequence of three *Achyranthes* species chloroplast genome are relatively conservative. Each of the three *Achyranthes* species chloroplast genome were predicted to encode 127 genes, including 82 protein-coding genes, 37 tRNA genes and 8 rRNA genes (Table 2). These genes classified to 17 groups according to their function. Additionally, the IRs regions contain 6 protein-coding and 7 tRNA genes, and the LSC and SSC region contain 67 and 11 protein-coding genes, respectively, meanwhile, the LSC and SSC region included 29 and one tRNA genes, respectively (Fig. 1).

**Table 2:** List of genes annotated in the *Achyranthes* chloroplast genomes.

Category	Group of genes	Name of genes
Self-replication	Large subunit of ribosomal proteins	rpl2*^,a^, 14, 16*, 20, 22, 23^*a*^*, 33, 36*
	Small subunit of ribosomal proteins	*rps2, 3, 4, 7*^*a*^, 8, 11, 12*^,a^, 14, 16*, 18, 19
	DNA-dependent RNA polymerase	rpoA, B, C1*, C2
	rRNA genes	*rrn16S*^*a*^*, rrn23S*^*a*^*, rrn4.5S*^*a*^*, rrn5S*^*a*^
	tRNA genes	trnA-UGC*^,a^, trnC-GCA, trnD-GUC, trnE-UUC, trnF-GAA, trnfM-CAU, trnG-UCC*, trnG-GCC, trnH-GUG, trnI-CAU, trnI-GAU*^,a^, trnK-UUU*, trnL-CAA, trnL-UAA*, trnL-UAG, trnM-CAU, trnN-GUU, trnP-UGG, trnQ-UUG, trnR-ACG, trnR-UCU, trnS-GCU, trnS-GGA, trnS-UGA, trnT-GGU, trnT-UGU, trnV-GAC, trnV-UAC*, trnW-CCA, trnY-GUA
Photosynthesis	Photosystem I	*psaA, B, C, I, J*
	Photosystem II	*psbA, B, C, D, E, F, H, I, J, K, L, M, N, T, Z,*
	NADH oxidoreductase	ndhA*, B*^,a^*, C, D, E, F, G, H, I, J, K*
	Cytochrome b6/f complex	petA, B*, D*, G, L, N
	ATP synthase	atpA, B, E, F*, H, I
	Rubisco	*rbcL*
Other genes	Maturase	*matK*
	Protease	clpP*
	Envelope membrane protein	*cemA*
	Subunit acetyl-CoA-carboxylase	*accD*
	c-Type cytochrome synthesis gene	*ccsA*
	Conserved open reading frames	*ycf1, 2*^a^, 3*, 4, 15

*Genes containing introns.

^a^Duplicated gene (genes present in the IR regions).

Totally, there were 15 intron-containing genes, containing five tRNA genes and ten protein-coding genes (Table 3). Then thirteen genes included one intron, and the remaining two genes (*ycf3* and *clpP*) included two introns of these 15 genes. The length of intron in *trnK-UUU* gene is largest, which was approximately 2,483 bp, and it is same to *A. longifolia* of *Achyranthes*, but there is a length of 2,480 bp in intron of *trnK-UUU* gene in *A. aspera*.

**Table 3:** Length of exons and introns in genes with introns in the *Achyranthes* chloroplast genome.

Species	Gene	Location	Exon I (bp)	Intron I (bp)	Exon II (bp)	Intron II (bp)	Exon III (bp)
*A. longifolia*	*trnK-UUU*	LSC	35	2,483	37		
	*trnL-UAA*	LSC	35	388	50		
	*trnV-UAC*	LSC	37	594	38		
	*trnI-GAU*	IR	42	940	35		
	*trnA-UGC*	IR	38	820	35		
	*rps12*^a^	LSC	26	538	232		114
	*rps16*	LSC	214	900	41		
	*atpF*	LSC	410	795	145		
	*rpl16*	LSC	402	986	9		
	*rpoC1*	LSC	1602	763	432		
	*ndhB*	IR	756	676	777		
	*ycf3*	SSC	153	756	228	785	126
	*petB*	LSC	6	768	642		
	*clpP*	LSC	228	623	292	842	71
	*petD*	LSC	8	783	475		
*A. bidentata*	*trnK-UUU*	LSC	35	2,483	37		
	*trnL-UAA*	LSC	35	388	50		
	*trnV-UAC*	LSC	37	593	38		
	*trnI-GAU*	IR	42	940	35		
	*trnA-UGC*	IR	38	820	35		
	*rps12*^a^	LSC	25	537	231		113
	*rps16*	LSC	214	900	41		
	*atpF*	LSC	410	743	145		
	*rpl16*	LSC	402	966	9		
	*rpoC1*	LSC	1602	763	432		
	*ndhB*	IR	756	676	777		
	*ycf3*	SSC	153	756	228	785	126
	*petB*	LSC	6	768	642		
	*clpP*	LSC	228	623	292	841	71
	*petD*	LSC	8	783	475		
*A. aspera*	*trnK-UUU*	LSC	35	2,480	37		
	*trnL-UAA*	LSC	35	388	50		
	*trnV-UAC*	LSC	37	594	38		
	*trnI-GAU*	IR	42	940	35		
	*trnA-UGC*	IR	38	820	35		
	*rps12*^a^	LSC	25	537	231		113
	*rps16*	LSC	214	900	41		
	*atpF*	LSC	410	790	145		
	*rpl16*	LSC	402	985	9		
	*rpoC1*	LSC	1602	762	432		
	*ndhB*	IR	756	676	777		
	*ycf3*	SSC	153	756	228	784	126
	*petB*	LSC	6	776	642		
	*clpP*	LSC	228	622	292	844	71
	*petD*	LSC	8	784	475		

^a^The *rps12* gene is a trans-spliced gene with the 5′ end located in the LSC region and the duplicated 3′ ends in the IR regions.

### Long repeat structure analysis

**Figure 2:**
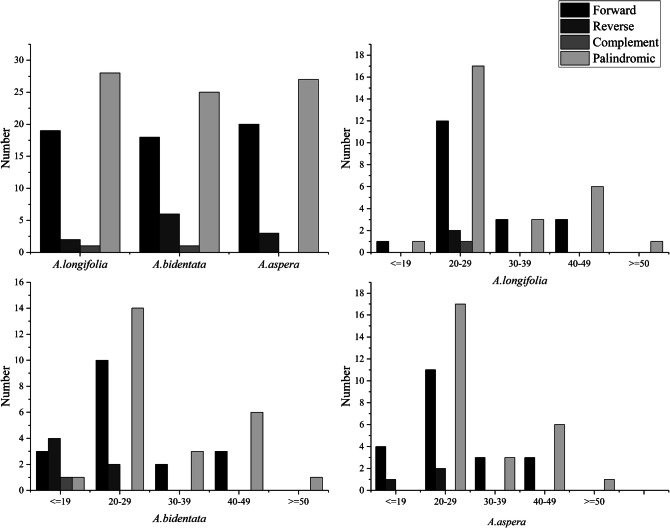
Analysis of repeated sequences in three *Achyranthes* chloroplast genomes.

In the 3 *Achyranthes* species chloroplast genome, there were 50 repeats were detected, which contained forward repeats, reverse repeats, complement repeats and palindromic repeats (Fig. 2). Then, there were 19 forward repeats, 2 reverse repeats, one complement repeats and 28 palindromic repeats in *A. longifolia* chloroplast genome. And there were 18 forward repeats, 6 reverse repeats, one complement repeats and 25 palindromic repeats in *A. bidentata* chloroplast genome. Then there were 20 forward repeats, 3 reverse repeats and 27 palindromic repeats in *A. aspera* chloroplast genome. However, there was no complement repeats in *A. aspera* chloroplast genome. These results also presented that it had a length about 20–29 bp in most forward repeats of three *Achyranthes* chloroplast genome. Then the length of most reverse repeats is below 19 bp, and the length of complement repeats is only below 19 bp, the length of most palindromic repeats was 20–29 bp in *A. bidentata* chloroplast genome. However, there was a different phenomenon in the *A. longifolia* and *A. aspera* chloroplast genome. In the *A. longifolia* chloroplast genome, the length of most reverse repeats and complement repeats were about 20–29 bp. Then the length of most reverse repeats was about 20–29 bp and there was no complement repeats in *A. aspera* chloroplast genome.

### Simple sequence repeat (SSR) analysis

**Figure 3:**
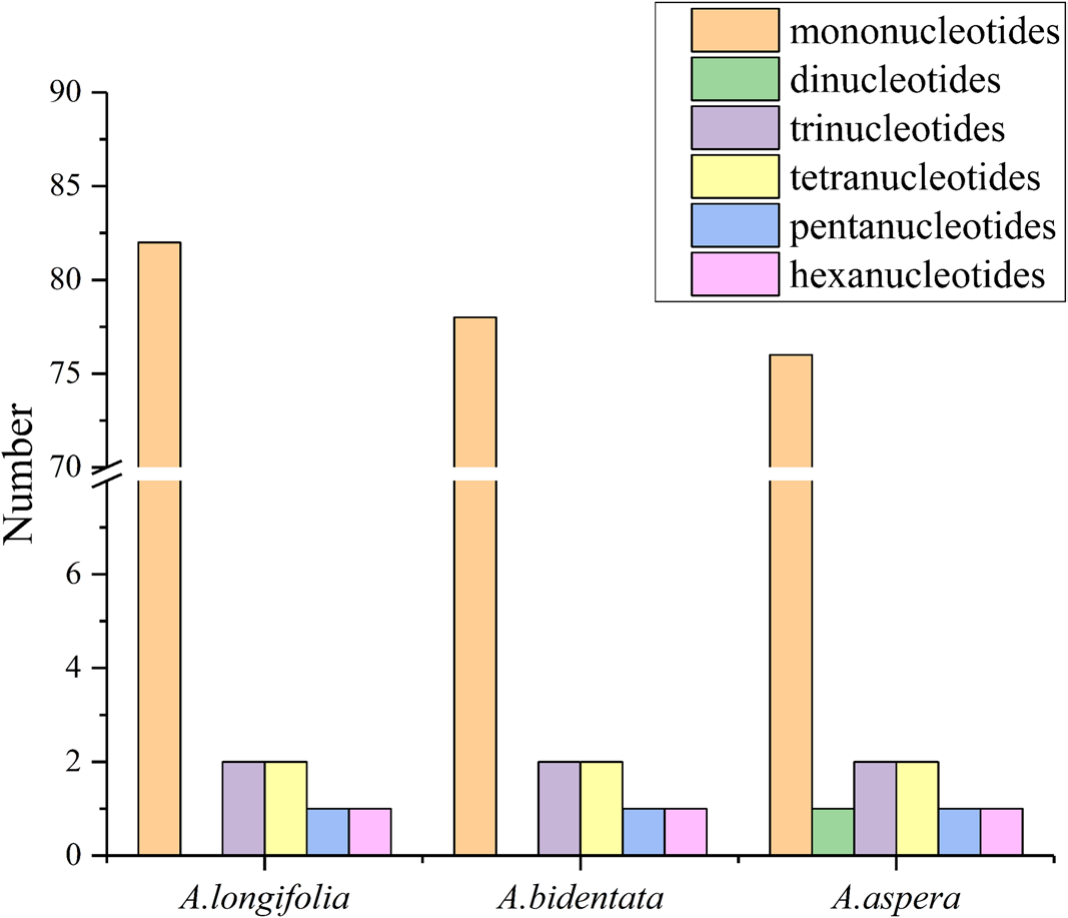
Analysis of simple sequence repeats (SSRs) in three *Achyranthes* chloroplast genomes.

Simple sequence repeats (SSR), it was known as a microsatellite, including 1–6 nucleotides, and it was widely distributed the genome. In our study, the SSRs were analyzed in the *Achyranthes* chloroplast genome (Fig. 3), and the numbers and distributions of the SSRs were very similar in the three chloroplast genomes, but there were some differences. The result of research revealed that there were 88, 84, 83 SSRs in *A. longifolia, A. bidentata, A. aspera* chloroplast genome, and there were 82, 78, 76 mononucleotides SSRs in *A. longifolia, A. bidentata, A. aspera*, respectively. However, there is only one dinucleotide occurred in *A. aspera.* The result showed that the number of mononucleotides SSRs is maximum of *A. longifolia* among these *Achyranthes* species, and the dinucleotides repeat content is the least in all species. According to the result, the high variability of SSRs in these chloroplast genomes provides strong value and evidence for molecular breeding and identification of medicinal plants.

### Comparative chloroplast genomic analysis in three *Achyranthes* species

In this study, the comparison of structure among three *Achyranthes* chloroplast genomes were performed. The result indicated that there was a length of 151,520 bp (*A. longifolia*), 151,284 bp (*A. bidentata*), 151,486 bp (*A. aspera*) in these *Achyranthes* chloroplast genome, and the length of IRs regions of *A. bidentata* is 25,150 bp, which has the same length with *A. longifoli*. And it had the smallest SSC region among these sequenced chloroplast genomes of *Achyranthes*.

**Figure 4:**
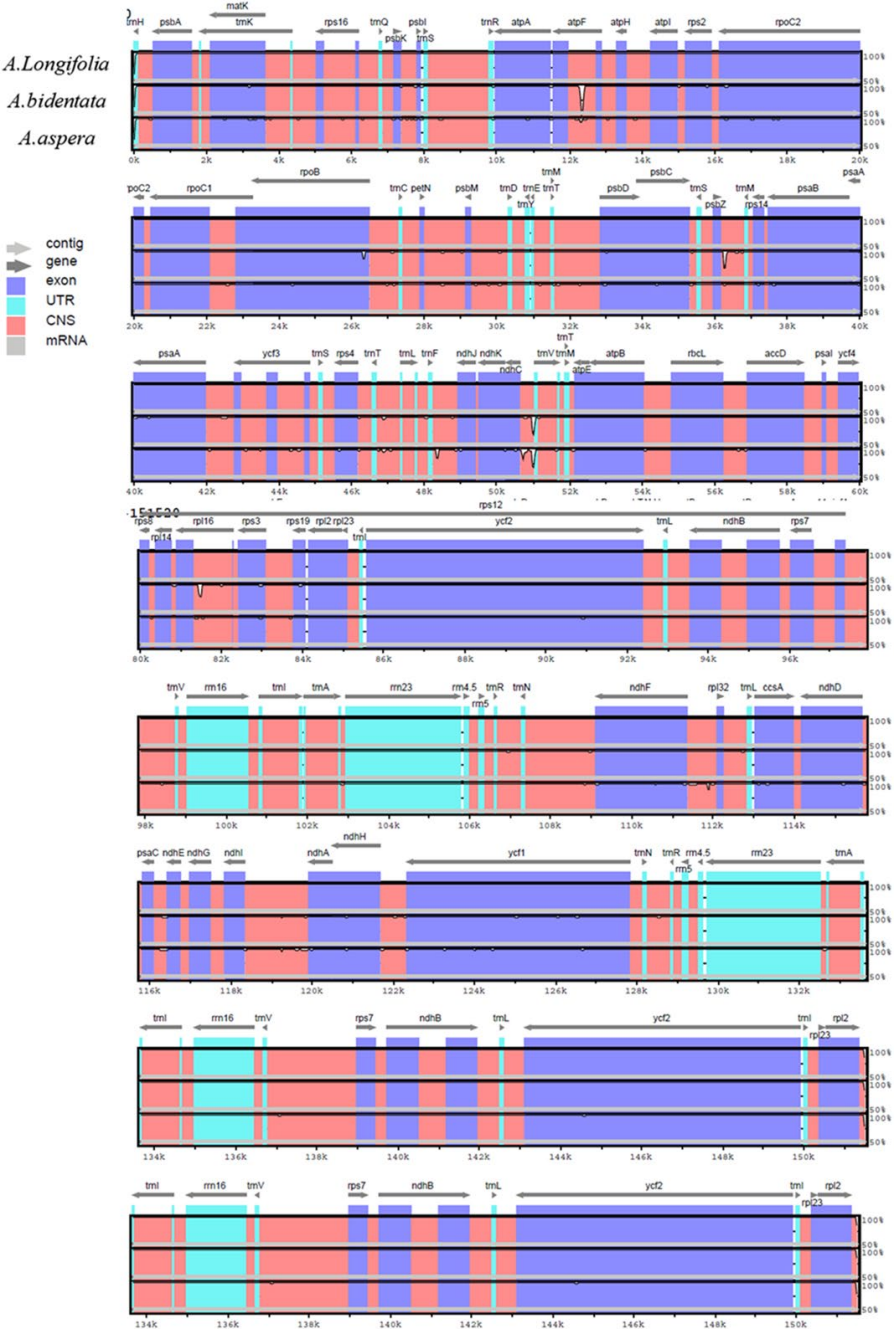
Comparison of the chloroplast genomes using mVISTA. Gray arrows and thick black lines above the alignment indicate gene orientation. Purple bars represent exons, blue bars represent untranslated regions (UTRs), pink bars represent noncoding sequences (CNS), gray bars represent mRNA, and white peaks represent differences of genomics. The y-axis represents the percentage identity.

In addition, to analysis the DNA sequences divergence of related species, other chloroplast DNAs was premeditated using mVISTA, and with the chloroplast genome of *A. bidentata* as a reference (Fig. 4). The results showed that the LSC and SSC regions were no more difference than a pair of IRs regions in length. Besides, the coding regions were less flexible than the noncoding regions, and the highly divergent regions was found in the intergenic spaces amongst these *Achyranthes* chloroplast genomes.

### IR contraction and expansion in the chloroplast genome of *Achyranthes*

**Figure 5:**
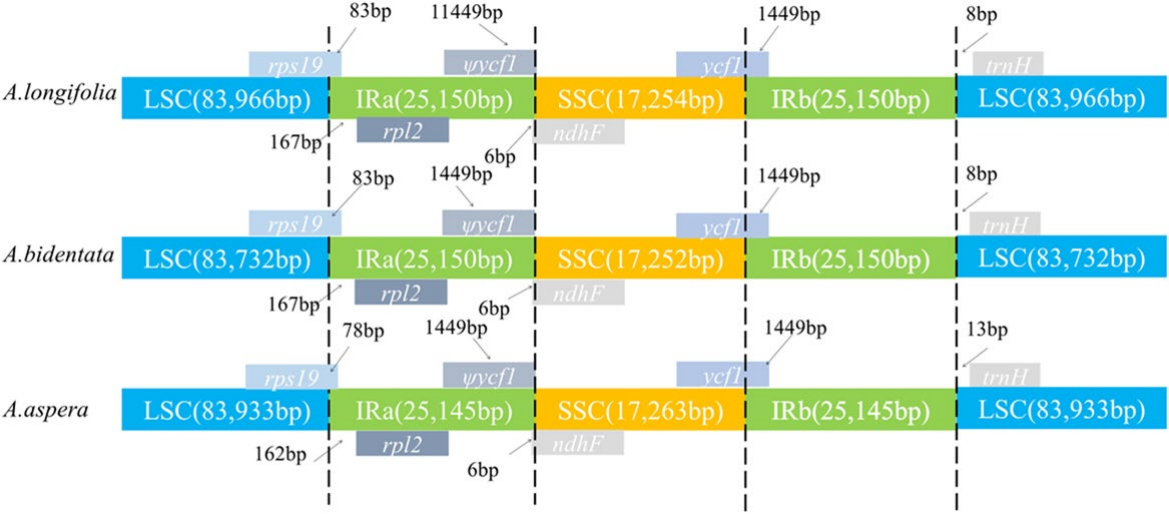
Comparison of border distance between adjacent genes and junctions of the LSC, SSC and two IR regions among the chloroplast genomes of three *Achyranthes* species. Boxes above or below the main line indicate the adjacent border genes. The figure is not to scale with respect to sequence length, and only shows relative changes at or near the IR/SC borders.

In our study, the detailed comparison of the IR-LSC and IR-SSC border structure among three species (*A. longifolia*, *A. bidentata*, *A. aspera*) was accomplished (Fig. 5). Findings revealed that the SSC/IRa connection was positioned in the *ndhF* region in three species of *Achyranthes* chloroplast genome, and extends a length of 6 bp into the IRa region in all three species. Meanwhile, the *rps19* gene was positioned in the LSC/IRa junction and extend a length (*A. longifolia*, 83 bp; *A. bidentata*, 83 bp; *A. aspera*, 78 bp) into the IRa region. Then the SSC/IRb junction was located in the *ycf1* region and extends a length (*A. longifolia*, 1449 bp; *A. bidentata*, 1449 bp; *A. aspera*, 1449 bp) into the IRb region in the chloroplast genome. In the intervening time, the *trnH* gene was generally present in the LSC region, and it had a length of 8 bp, 8 bp, 13 bp from the LSC/IRb border in the chloroplast genome of *A. longifolia, A. bidentata, A. aspera*, respectively.

### Phylogenetic analysis

**Figure 6:**
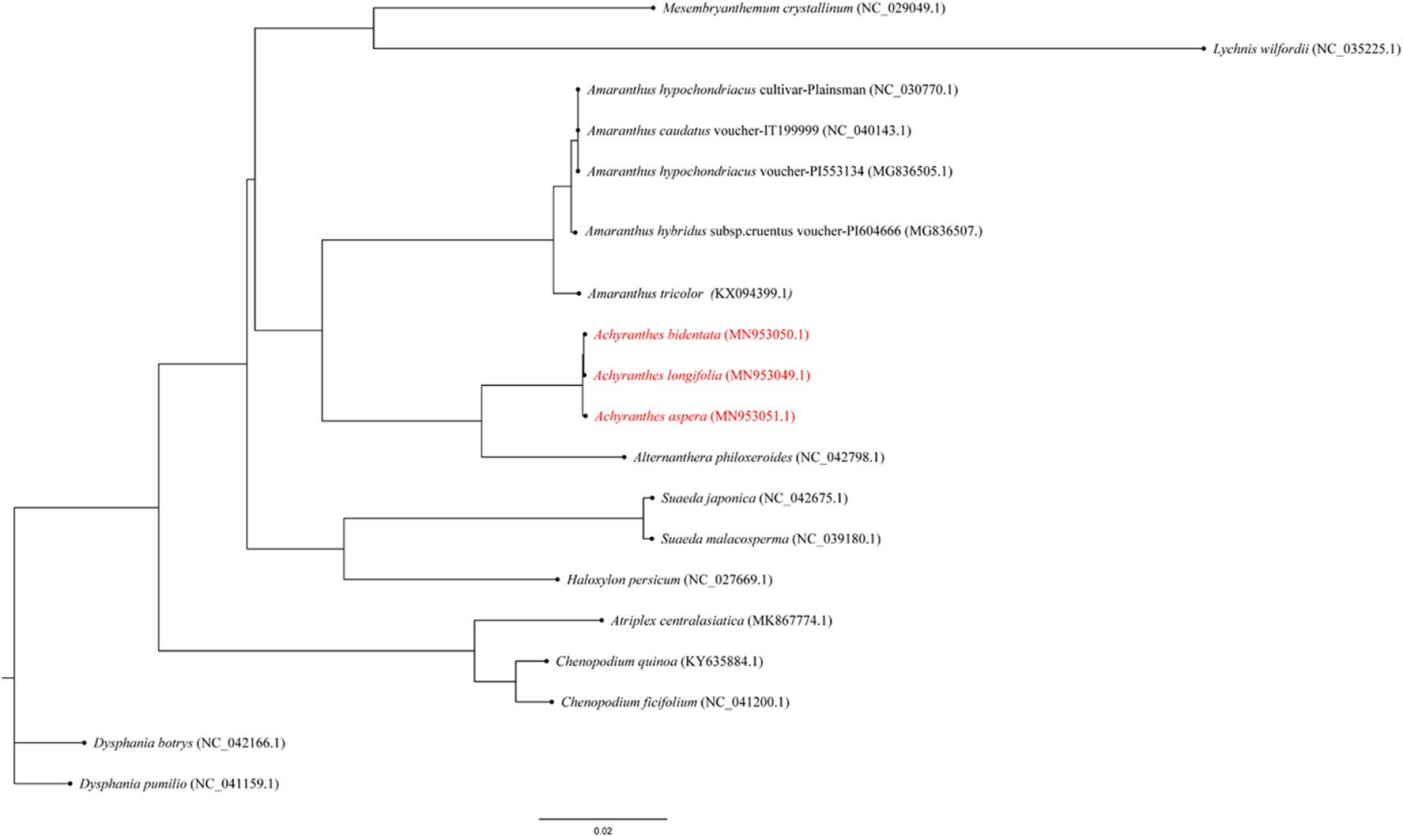
ML phylogenetic tree reconstruction including 19 species based on all chloroplast genomes.

Now there are more and more studies using complete chloroplast genome sequences to evaluate phylogenetic relationships between medicinal plants. Understanding the phylogenetic relationships between *Achyranthes* species and other Amaranthaceae could provide favorable guidance into the related angiosperm species. In this study, in order to analyze the phylogenetic relationships of *Achyranthes* species, the chloroplast genome sequences among 16 angiosperm species form NCBI (Fig. 6). On the basis of chloroplast genome date, the phylogenetic tree was built by the method of maximum likelihood (ML). The chloroplast genomes of 19 Centrospermae species were compared. The result showed that each genus and family of these species are divided into a taxonomic division, and constituted a monophyletic group in Centrospermae. The ML phylogenetic tree showed that there were divided into 13 clades among these analyzed species, and the result demonstrated *Achyranthes* is a monophyletic group, three species of *Achyranthes* belong to the one branch, which is separated from other genus of Amaranthaceae. In this taxon, *A. bidentata* had a strong sister relationship with *A. longifolia. A. bidentata* and *A. longifolia* were clustered together, the three *Achyranthes* species had the same origin, then the gunes of *Achyranthes* is the closest relative to *Alternanthera*, and that forms a group with *Alternanthera philoxeroides*. In addition, these results also provided effective evidence that the evolution of *A. bidentata* and *A. longifolia* occurred in the same direction.

## Discussion

In this study, the chloroplast genome of three *Achyranthes* species was analyzed, the results showed that the three *Achyranthes* species in this study were content with the characteristics of angiosperms both in structure and content. The typical quadripartite structure of the *Achyranthes* chloroplast genome are consistent with the characteristics of the chloroplast genome in medicinal angiosperms^32^. The GC content was lower than the AT content in the chloroplast genome of three *Achyranthes* species, and all these proved that there was no significant difference in chloroplast genomes among three *Achyranthes* species. The phenomenon was universal in other angiosperms chloroplast genomes^33–35^. And the results also showed that the GC content the highest in the IR regions, which may be caused by the presence of large amounts of rRNA in the IR regions. The specific reasons will require further research. And the results of coding regions and the highly divergent regions amongst these *Achyranthes* chloroplast genomes, were also found in other plants chloroplast genomes^36–39^.

The length of exons and introns in genes were important information in plant chloroplast genome. In this study, the results showed that there were one gene (*rps12*) included three exons, and two genes (*ycf3* and *clpP*) included two introns in three *Achyranthes* chloroplast genome. The *rps12* gene is a trans-spliced gene with the 5′ end located in the LSC region and duplicated 3′ ends in the IRs regions^40^. Moreover, it has been reported that *ycf3* is a gene closely related to photosynthesis^41, 42^. Consequently, the attainment of *ycf3* gene will contribute to the further investigation of chloroplast in *Achyranthes.* The *ycf1* gene also played a vital role in the chloroplast genome, there were the related reports on gene function of *ycf1*, these reports revealed *ycf1* is an important pseudogene for the chloroplast genome variation and encoding of *Tic214* in plants^43, 44^.

According to the previous reports, these introns played a vital role in the regulation of the gene expression^45^, which could adjust the level of the gene expression in a special spatiotemporal^46, 47^. Moreover, we found that some phenomenon in the chloroplast genomes, such as the intron or gene losses^48–50^, and the regulating function of intron have been found in many plants chloroplast genome^51, 52^. However, now there were no related research on the introns regulation mechanism of *Achyranthes*. Therefore, we could attain more useful information through the further studies of introns in the chloroplast genomes. The information of chloroplast genome could provide important theoretical basis for plant resource identification, especially medicinal plants.

Long repeats and the SSRs of the chloroplast genome were the vital information for identification of plant germplasm resources and molecular markers. Studies have shown that there are more than 30 bases of 14 repeats in *S.miltiorrhiza* chloroplast genome, similar, the repeats of ≥ 30 bases were 16, 15 and 16 in the chloroplast of *A. longifolia*, *A. bidentate and A. aspera,* respectively. The results of this study show that genes with long repeat sequences may be very suitable for genetic marker identification of related species, and the specific role needs to be proved by subsequent studies.

SSRs play a vital role in the chloroplast genomes. Due to its extreme variability, it was used to genetic research^53–55^. Previous report showed that the SSR was commonly distributed the genome, and the SSR was widely used to the genetic population structure and maternity analysis because of its unique uniparental in inheritance. Previous studies have shown that the mononucleotides were the most abundant repeats in *A.formosae*, and there was the same phenomenon in the three *Achyranthes* chloroplast genome. Therefore, the study of the chloroplast genome SSRs will greatly promote the investigation of species identification, genetic diversity and evolutionary process in *Achyranthes*^56, 57^.

Previous research had shown that IRs regions were the most conserved regions in the chloroplast genome^19^. Its contraction and expansion at the borders is a general evolutionary event, and which represent the dominant reason for the size variation and rearrangement of the chloroplast genome^58–60^. There were many reports that the chloroplast gene had a conservation order in most land plants, but there were also reports that many sequences were rearranged in the chloroplast genomes of most plant species, then the IR contraction and expansions with inversions, the inversions in the LSC region and the re-inversion in the SSC region were included^61–63^, and some reports showed that the extensive rearrangements in the chloroplast genome of *Trachelium caeruleum* are associated with repeats and tRNA genes^64^. Because of the sequence rearrangements that modification of chloroplast genome structure in associated species may be related to the plant genetic diversity information, so it can be used for molecular identification and evolutionary research^65^.

With the continuous development of next generation sequencing technology, especially the application of second-generation sequencing technology, chloroplast genome sequencing has become simpler and easier than first generation sequencing. Moreover, at present, more and more researches have used the complete CP genome sequence to evaluate the phylogenetic relationship between angiosperms. In this study, The ML phylogenetic tree showed that there were divided into 13 clades among these analyzed species, and the results showed that there was a strong sister relationship between *A. bidentate* and *A. longifolia*. The chloroplast genomes were vital genomic resources for the reconstruction of precise high-resolution phylogenies^66^. As a member of the Amaranthaceae family, *Achyranthes* species contained vital genetic resources for the evolution and development of other species^67, 68^. The *Achyranthes* species and *Alternanthera philoxeroides* come from a monophyletic group, which is consistent with the results of Park^30^. However, the *A.bidentate* formed a group with *Cyathula capitata* and with 100% bootstrap in the research of Li^31^. Combined with our phylogenetic analysis and Li's research results, it is speculated that there may be a far-reaching relationship between *A.bidentata* of Hubei and *A.bidentata* from other regions, indicating that geographic isolation may have a greater impact on the interspecific relationship of *Achyranthes*. And in this study, we found that in the Amaranthaceae, each genus is basically clustered independently, indicating that there was a good monophyletic separation in this family.

At present, there are three species of *Achyranthes* species in China, and most of the studies are concentrated on *A. bidentate* and *A. aspera* in the world. Some studies have shown that the combined extract of *Lycii Radicis Cortex* and *A. japonica* had the effect of anti-osteoporosis^69^, in addition, it was also found that tannins isolated from leaf callus cultures of *A.aspera* and *O.basilicum* had the ability of anti-inflammatory and promoting wound healing^70^. Then some studies have also shown that the quality of chicken can be affected by adding the extract of *A. japonica* to chicken feed^71^. Therefore, it is speculated whether the addition of *A. japonica* extract to human diet will also affect the body muscle quality, which needs further research to prove. All these studies provided theoretical support for the research and development of *Achyranthes* in the future.

Now it has been shown that chloroplast genome can be used as super barcode to identify plant species^72^. According to our phylogenetic analysis of the chloroplast genome of three *Achyranthes* species, we speculated that the chloroplast genome of *Achyranthes* might be an important marker for species identification. Further research is needed to study this conjecture. The study results are of great value to the evaluation of genetic diversity and phylogenetic research of *Achyranthes* in the future. However, unfortunately, our study did not fully understand the relationship between genera. In addition, our phylogenetic study only is based on the chloroplast genome. If we want to fully understand the phylogeny of species in Amaranthaceae and even Centrospermae, we may need to analyze the nuclear genes of plants, and more genera should be included in the future. Nevertheless, our phylogeny research provided valuable resources for the classification, phylogeny and evolutionary history of *Achyranthes*.

## Conclusions

*Achyranthes* L. is the extremely important medicinal plant. The chloroplast genome contains a large amount of available genetic information. At present, there is almost no research on the chloroplast genome of *Achyranthes genus* around the world. Consequently, it is extremely important to explore the genetic evolution and phylogeny by studying the genetic information of chloroplast genome of *Achyranthes.* In this study, the chloroplast genome sequencing of the *three Achyranthes* species was performed by next generation sequencing technology, the complete chloroplast genome sequence was obtained of the *Achyranthes.* This is an important finding about complete chloroplast genome of *Achyranthes* in China*.* The result revealed that the chloroplast genome of *A. bidentata* has a highly conserved structure, it was similar to angiosperms. Then we also determined the SSR, protein-coding gene sequence and repeated sequences, the phylogenetic analysis shows that there was a closer relationship between *A. bidentata* and *A. longifolia*. These results will offer the correlative supportable evidences and lay a solid foundation for the development of chloroplast genome of Amaranthaceae plants.

## Materials and methods

### Materials and DNA extraction

Fresh materials leaves of the *A. bidentate* were collected from Wuzhi County, Jiaozuo City, Henan Province of China (N35° 04′ 43.03″, E113° 24′ 7.69″), and *A. aspera* and *A. longifolia* were obtained from the field in Tongbai County, Nanyang City, Henan province in China (N32° 38′ 56.23″, E113° 43′ 50.46″). The fresh leaves of plant materials were quickly frozen with liquid nitrogen immediately after picking and cleaning, and kept in low temperature and dark. Total genomic DNA of them were extracted with Plant Genomic DNA Kit (TIANGEN, BEIJING) and the integrity of the extracted total genomic DNA was detected by 1% agarose gel electrophoresis, the total concentration of the extracted DNA was estimated by an ND-2000 spectrometer (Nanodrop Technologies, Wilmington, DE, USA)^73^, then the qualified samples were selected for subsequent experiments.

### Chloroplast genome sequencing and assembly, annotation and structure

After the sample are qualified, the sequencing library was constructed by means of purified the fragment, repaired the terminal, added with A in 3′ segment, connected with sequencing connector, PCR amplification, etc. Primers used for assembly validation can be found as Supplementary Table S2 online. The library type is the DNA small fragment library of 250 bp, then sequencing was performed by an Illumina Hiseq X Ten platform pair-end sequencing method. Firstly, the low quality and redundant reads (Q < 20) were trimmed using Skewer-0.2.2^74^, then the CP-like reads were extracted from those clean reads in comparison using the BLAST searches^75^ and these CP-like reads be used for sequence assembly with SOAPdenovo-2.04^76^, then to verifying the four junction regions between the IR regions and the LSC/SSC region, PCR amplification was performed. At last, resulting in a complete chloroplast genome sequence of *A. bidentata.* Gene annotation of the three *Achyranthes* species chloroplast genome were performed using the CpGAVAS with default parameters^77^, the physical chloroplast genome map was drawn using the OGDRAWv1.2 program with default parameters or subsequent manual editing^78^.

### Long-repeats, simple sequence repeats and genome comparison

The REPuter was used to detect the forward (inverted) repeats. The simple sequence repeats (SSRs) in the chloroplast genome of *A. bidentata* were identified by using Phobos version 3.3.12^79^. The chloroplast genomic sequences alignment was carried out using the clustalw2^80^. The mVISTA^81^ program in the Shuffle-LAGAN mode^82^ was used to compare the whole chloroplast genome of *A. bidentata.* with *A. longifolia* chloroplast genomes and *A. aspera* chloroplast genomes.

### Phylogenetic analysis

In order to investigate the phylogenetic position of *A.bidentata* within Amaranthaceae lineages, then 16 complete chloroplast (CP) genome sequences were downloaded from the NCBI Organelle Genome database. Maximum likelihood (ML) analyses were conducted using the whole cp genome sequences. The cp genome sequences alignment was carried out using the clustalw2. The best model was determined by modeltest-ng-0.1.6 software with default parameters^83^, ML analyse was performed using RAxML-NG v0.9.0^84^ based on Linux edition using default parameters. The parameters were GTR {0.901389/1.745760/0.433440/0.576338/1.701638/1.000000} + FU {0.323117/0.177967/0.173322/0.325594} + G4m {0.329075}, noname = 1–244,745.

## Supplementary information

Supplementary file 1

Supplementary file 2

## Data Availability

The original sequencing datas have been submitted to the NCBI database and got the GenBank accession number MN953049 (*A. longifolia*), MN953050 (*A. bidentata*), MN953051 (*A. aspera*).
