# Supplementary material for: Comparing and phylogenetic analysis chloroplast genome of three Achyranthes species
Source: Sci Rep. 2020 Jul 2;10:10818. doi: 10.1038/s41598-020-67679-y (PMC7331806; doi:10.1038/s41598-020-67679-y)
Supplement: Supplementary file 1 [file 41598_2020_67679_MOESM1_ESM.docx]

**Comparing and Phylogenetic Analysis Chloroplast Genome of three** ***Achyranthes* Species**

**Jingya Xu^1,2,3,4^, Xiaofeng Shen^3,4^, Baosheng Liao^3^, Jiang Xu*^3^, Dianyun Hou*^1,2^**

^1^Agricultural College, Henan University of Science and Technology, Luoyang, China. ^2^The Luoyang Engineering Research Center of Breeding and Utilization of Dao-di Herbs, Luoyang, China. ^3^Institute of Chinese Materia Medical, China Academy of Chinese Medical Sciences, Beijing, China; ^4^These authors contributed equally: Jingya Xu and Xiaofeng Shen. *email: jxu@icmm.ac.cn;dianyun518@163.com

Table S 1. Sequence alignment result and partial DNA peak map

| Species | Sequence name | Sequence alignment result | Partial DNA peak map |
| --- | --- | --- | --- |
| *A.bidentata* | LSC-IRb | Note 1-1 | Note1-2 |
| *A.bidentata* | IRb-SSC | Note 1-3 | Note 1-4 |
| *A.bidentata* | SSC-IRa | Note 1-5 | Note 1-6 |
| *A.bidentata* | IRa-LSC | Note 1-7 | Note 1-8 |
| *A.longifolia* | LSC-IRb | Note 2-1 | Note 2-2 |
| *A.longifolia* | IRb-SSC | Note 2-3 | Note 2-4 |
| *A.longifolia* | SSC-IRa | Note 2-5 | Note 2-6 |
| *A.longifolia* | IRa-LSC | Note 2-7 | Note 2-8 |
| *A.aspera* | LSC-IRb | Note 3-1 | Note 3-2 |
| *A.aspera* | IRb-SSC | Note 3-3 | Note 3-4 |
| *A.aspera* | SSC-IRa | Note 3-5 | Note 3-6 |
| *A.aspera* | IRa-LSC | Note 3-7 | Note 3-8 |

Note 1-1: Sequence alignment result of LSC-IRb junction sequence of *A. bidentata*


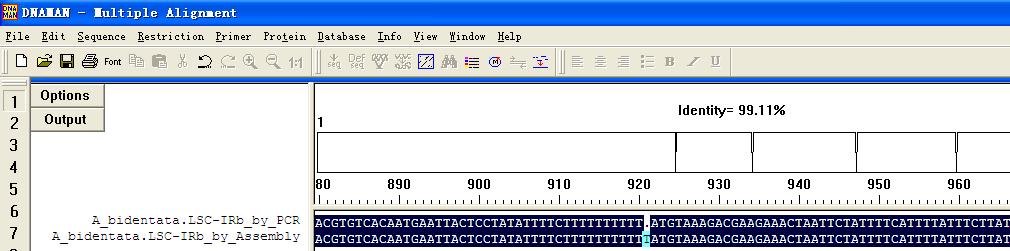


Note 1-2: Partial DNA peak map of LSC-IRb junction sequence of *A. bidentata*


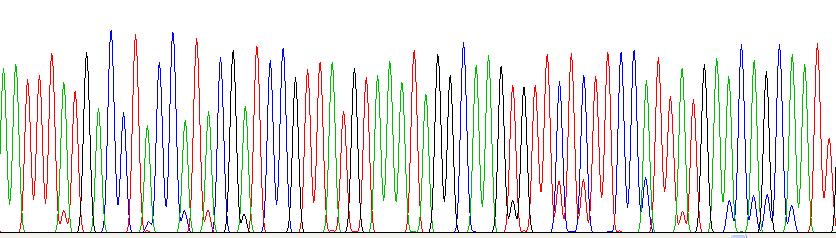

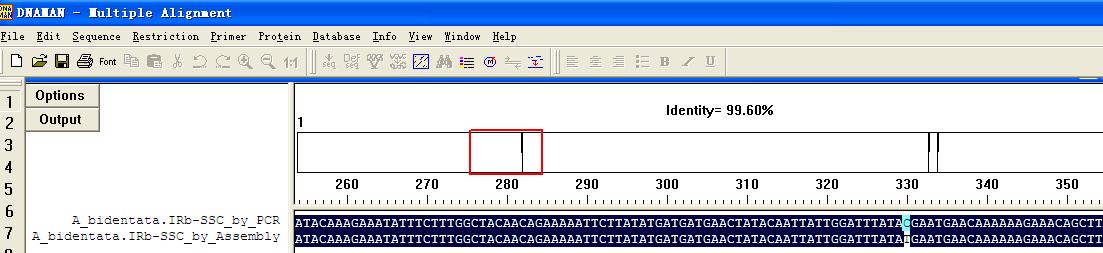


Note 1-3: Sequence alignment result of IRb-SSC junction sequence of *A. bidentata*


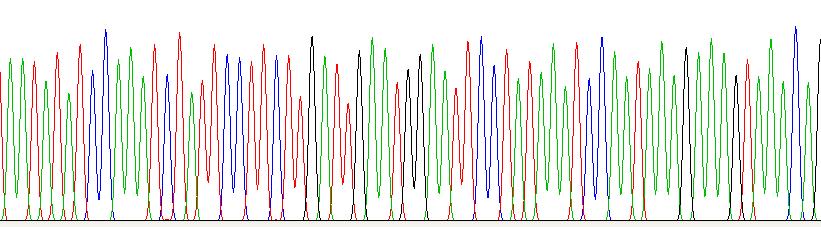


Note 1-4: Partial DNA peak map of IRb-SSC junction sequence of *A. bidentata*

Note 1-5: Sequence alignment result of SSC-IRa junction sequence of *A. bidentata*


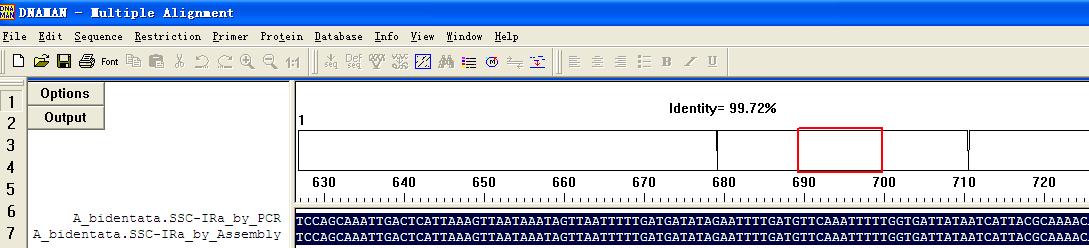


Note 1-6: Partial DNA peak map of SSC-IRa junction sequence of *A. bidentata*


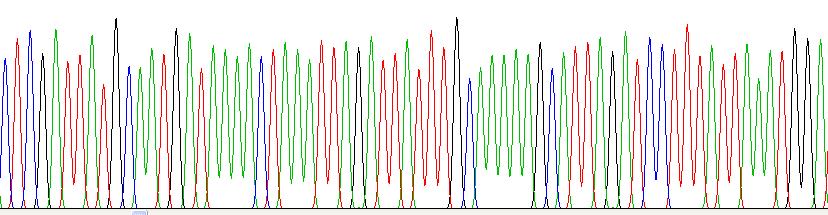

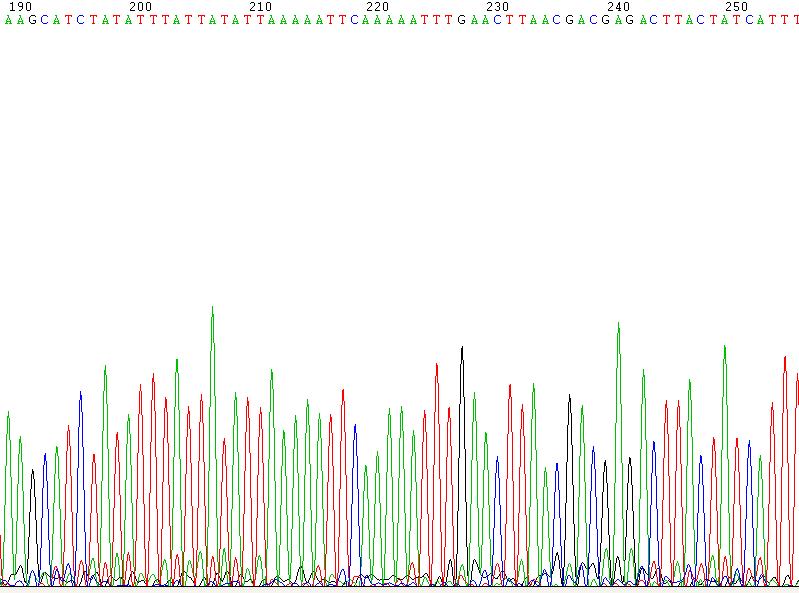


Note 2-2: Partial DNA peak map of LSC-IRb junction sequence of *A.longifolia*


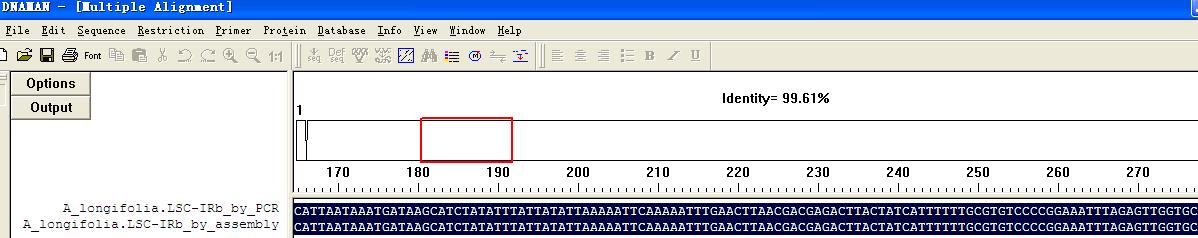


Note 2-1: Sequence alignment result of LSC-IRb junction sequence of *A.longifolia*


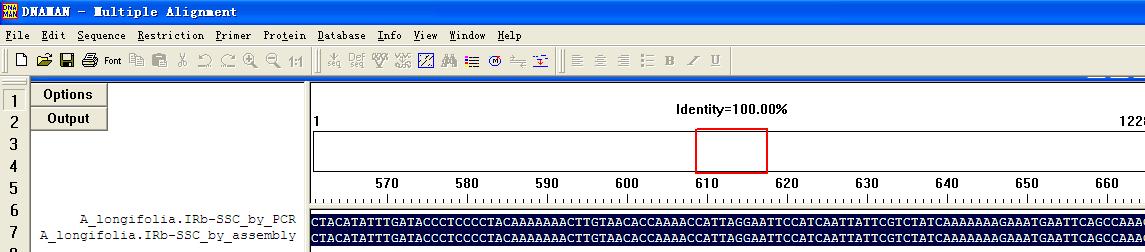


Note 2-3: Sequence alignment result of IRb-SSC junction sequence of *A.longifolia*

Note 2-4: Partial DNA peak map of IRb-SSC junction sequence of *A.longifolia*


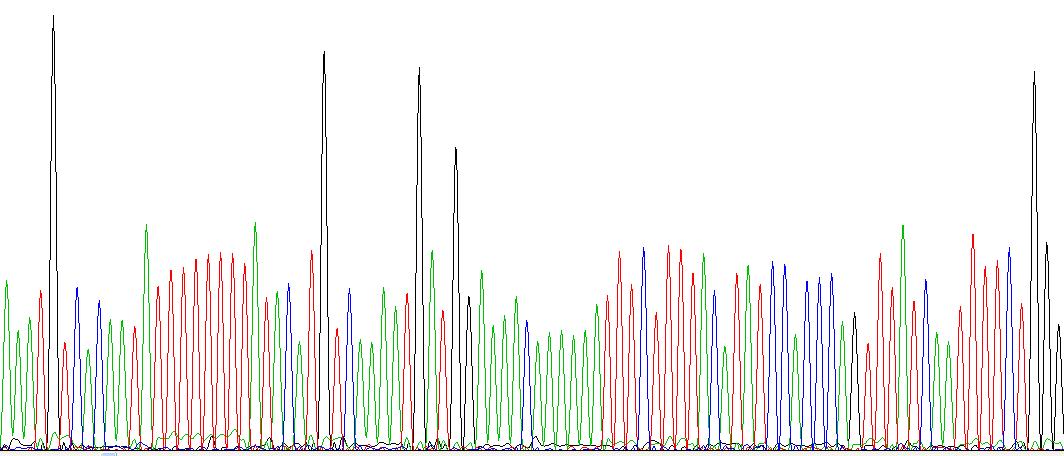


Note 1-7: Sequence alignment result of IRa-LSC junction sequence of *A. bidentata*


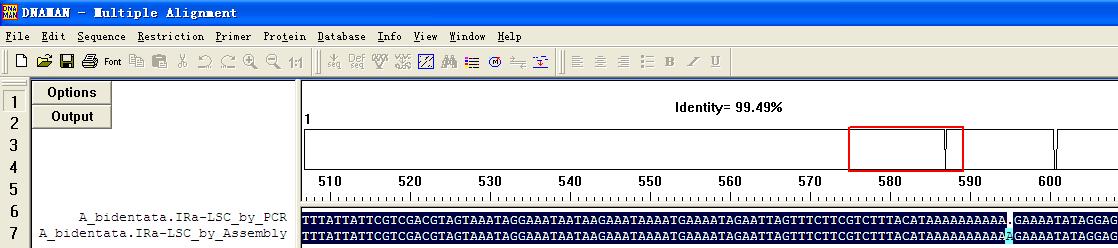


Note 1-8: Partial DNA peak map of IRa-LSC junction sequence of *A. bidentata*


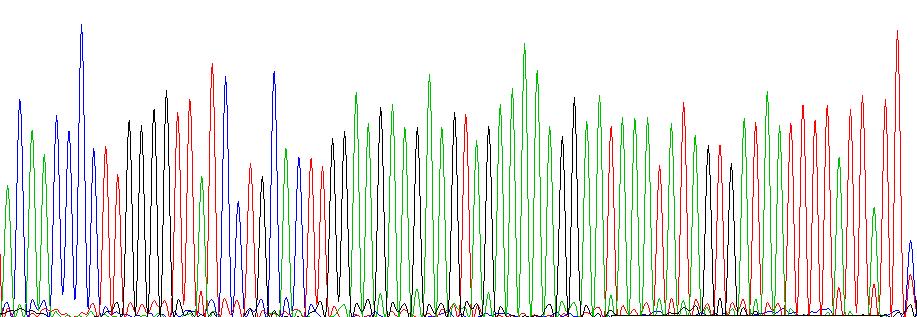


Note 2-5: Sequence alignment result of SSC-IRa junction sequence of *A.longifolia*


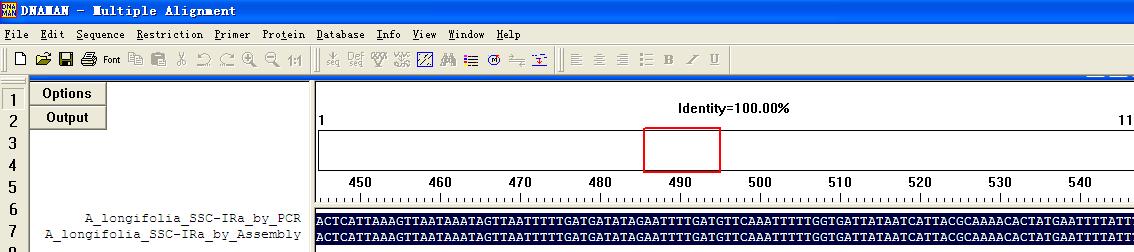


Note 2-6: Partial DNA peak map of SSC-IRa junction sequence of *A.longifolia*


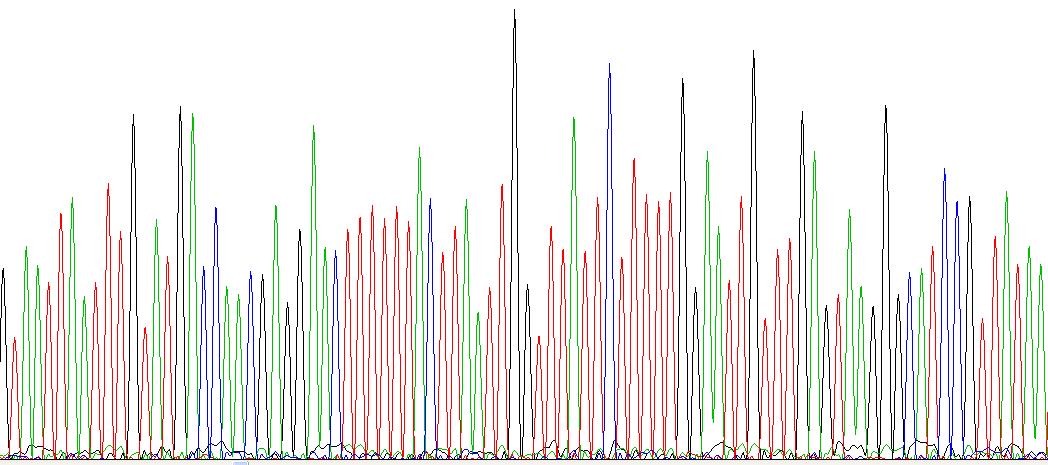

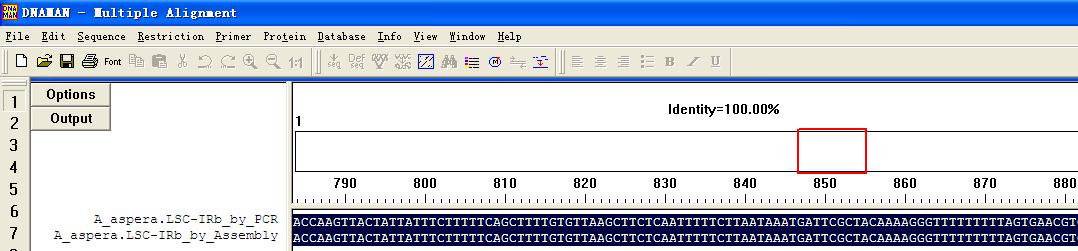


Note 3-1: Sequence alignment result of LSC-IRb junction sequence of *A.aspera*


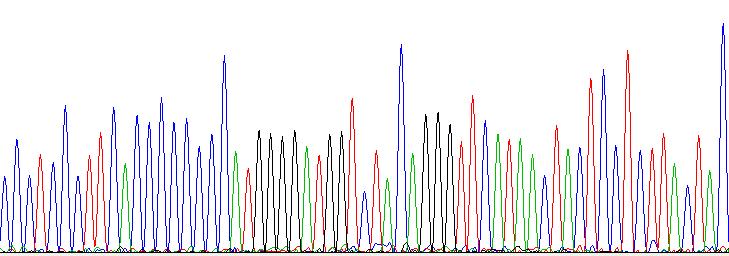


Note 3-2: Partial DNA peak map of LSC-IRb junction sequence of *A.aspera*


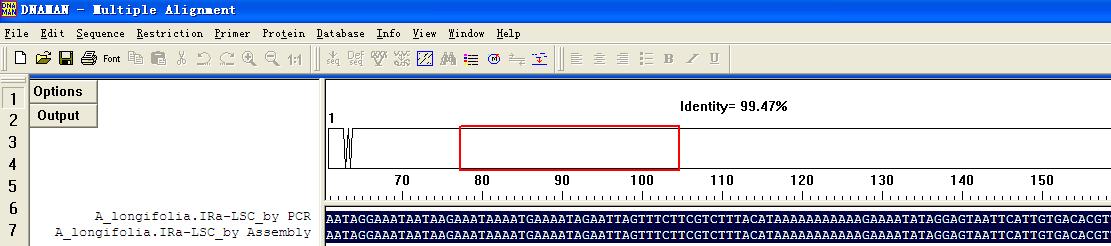


Note 2-7: Sequence alignment result of IRa-LSC junction sequence of *A.longifolia*


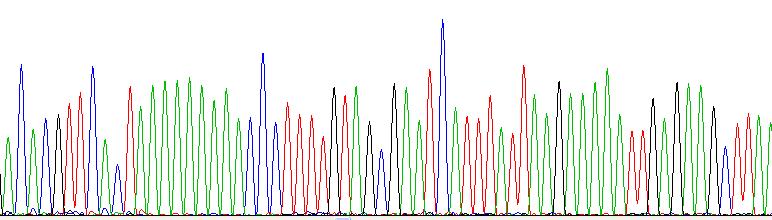


Note 2-8: Partial DNA peak map of IRa-LSC junction sequence of *A. longifolia*

Note 3-5: Sequence alignment result of SSC-IRa junction sequence of *A.aspera*


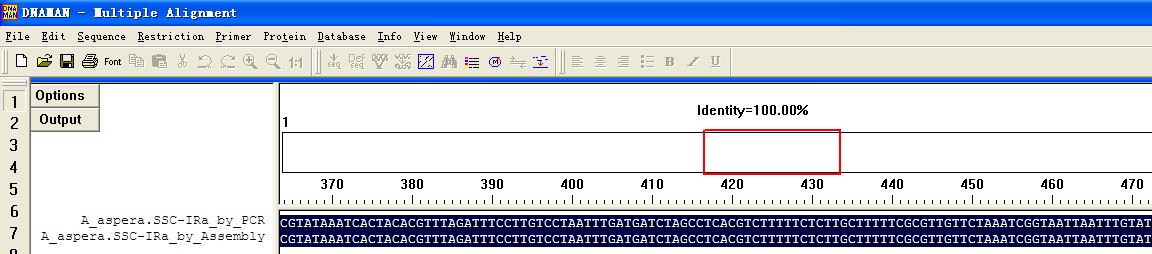

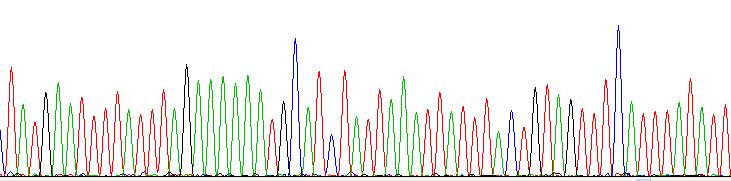


Note 3-6: Partial DNA peak map of SSC-IRa junction sequence of *A.aspera*


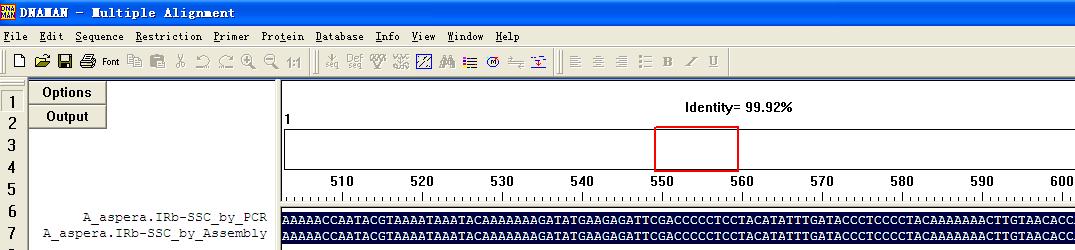


Note 3-3: Sequence alignment result of IRb-SSC junction sequence of *A.aspera*


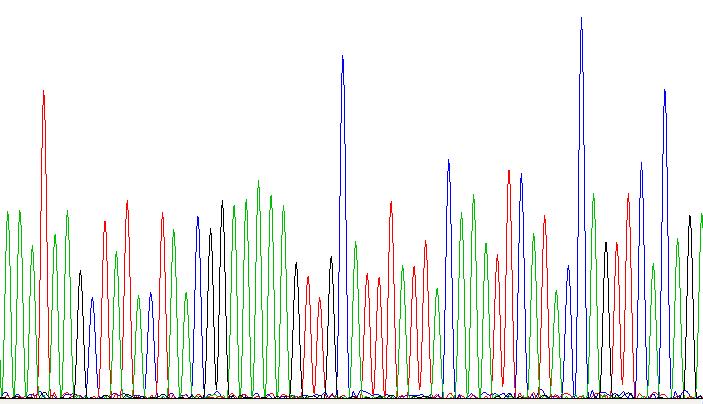


Note 3-4: Partial DNA peak map of IRb-SSC junction sequence of *A.aspera*

Note 3-7: Sequence alignment result of IRa-LSC junction sequence of *A.aspera*


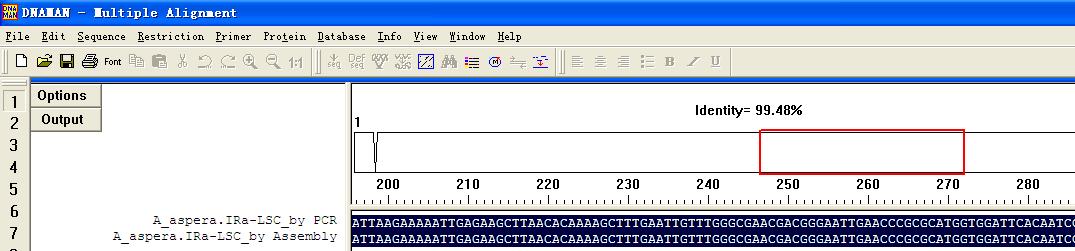


Note 3-8: Partial DNA peak map of IRa-LSC junction sequence of *A.aspera*


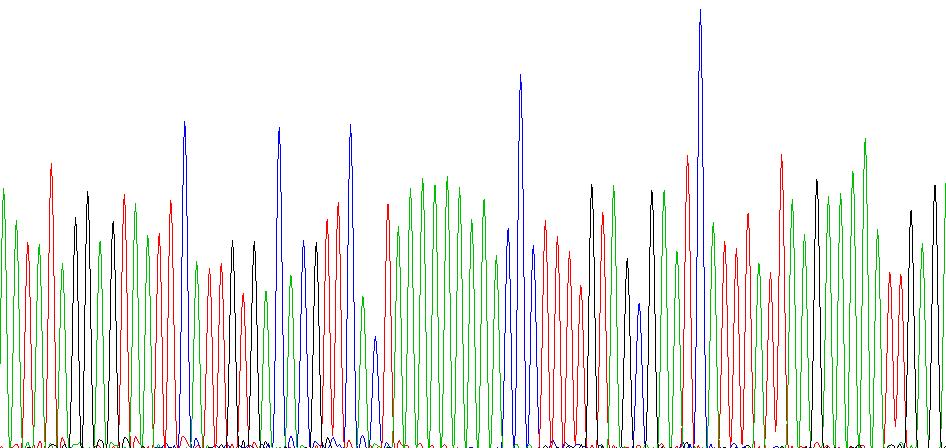


Note 2-1: Sequence alignment result of LSC-IRb junction sequence of A.longifolia

Note 2-2: Partial DNA peak map of LSC-IRb junction sequence of A.longifolia

Note 2-2: Partial DNA peak map of LSC-IRb junction sequence of A.longifolia
