# Supplementary material for: Comparing and phylogenetic analysis chloroplast genome of three Achyranthes species
Source: Sci Rep. 2020 Jul 2;10:10818. doi: 10.1038/s41598-020-67679-y (PMC7331806; doi:10.1038/s41598-020-67679-y)
Supplement: Supplementary file 2 [file 41598_2020_67679_MOESM2_ESM.docx]

**Comparing and Phylogenetic Analysis Chloroplast Genome of three** ***Achyranthes* Species**

**Jingya Xu^1,2,3,4^, Xiaofeng Shen^3,4^, Baosheng Liao^3^, Jiang Xu*^3^, Dianyun Hou*^1,2^**

^1^Agricultural College, Henan University of Science and Technology, Luoyang, China. ^2^The Luoyang Engineering Research Center of Breeding and Utilization of Dao-di Herbs, Luoyang, China. ^3^Institute of Chinese Materia Medical, China Academy of Chinese Medical Sciences, Beijing, China; ^4^These authors contributed equally: Jingya Xu and Xiaofeng Shen. *email: jxu@icmm.ac.cn;dianyun518@163.com

Table S2. Primers used for assembly validation

| Species | Primer | Sequence (5’>3’) | Amplicon Size (bp) |
| --- | --- | --- | --- |
| A.bidentata | LSC-IRb | AATAGAATCGGAAACGGTAGAATAAA | 1002 |
|  |  | CTTGGAAGAAGAAGTAGAAAAAGGAA |  |
| A.bidentata | IRb-SSC | GAATGGAAAGGACAAAACAAAGGATG | 1226 |
|  |  | ATGGGGTAAAGAAGGATTGAAAAGGA |  |
| A.bidentata | SSC-IRa | CTTGTCTTTGTTTTTCTTTTTTTTCC | 1065 |
|  |  | TCTTTTACATATCCACCCAGTTTATC |  |
| A.bidentata | IRa-LSC | TACACTATGGGGATGGTGAGAAGAG | 791 |
|  |  | CAAAGGCAAGTACAAGTAAAGAACT |  |
| A.longifolia | LSC-IRb | CTCATAGGAACGCCCACGAATCTG | 1335 |
|  |  | TGGTAAAGGTCGTAATGCCAGAGG |  |
| A.longifolia | IRb-SSC | CTCATAGGAACGCCCACGAATCTG | 951 |
|  |  | TGGTAAAGGTCGTAATGCCAGAGG |  |
| A.longifolia | SSC-IRa | GTCTAAAAAAAGTGGGGAATGCG | 1137 |
|  |  | TGGAAAGGACAAAACAAAGGATG |  |
| A.longifolia | IRa-LSC | GGAAGAAGAAGTAGAAAAAGGAATAA | 376 |
|  |  | ATCAAAGGCAAGTACAAGTAAAGAAC |  |
| A.aspera | LSC-IRb | TAATAGAATCGGAAACGGTAGAATAAA | 1433 |
|  |  | ACATAACATAGAAATCACACTTGGAAA |  |
| A.aspera | IRb-SSC | GAATGGAAAGGACAAAACAAAGGA | 1226 |
|  |  | TGGGGTAAAGAAGGATTGAAAAGG |  |
| A.aspera | SSC-IRa | TTTTCTTGTCTTTGTTTTTCTTTT | 813 |
|  |  | ATATCCACCCAGTTTATCAATTTT |  |
| A.aspera | IRa-LSC | TTGGAAGAAGAAGTAGAAAAAGGAA | 379 |
|  |  | AATCAAAGGCAAGTACAAGTAAAGA |  |
